# Effect of Accelerometer Cut-Off Points on the Recommended Level of Physical Activity for Obesity Prevention in Children

**DOI:** 10.1371/journal.pone.0164282

**Published:** 2016-10-10

**Authors:** Aleš Gába, Jan Dygrýn, Josef Mitáš, Lukáš Jakubec, Karel Frömel

**Affiliations:** Faculty of Physical Culture, Palacký University Olomouc, třída Míru 117, 771 11, Olomouc, Czech Republic; Vanderbilt University, UNITED STATES

## Abstract

There is no general consensus regarding which accelerometer cut-off point (CoP) is most acceptable to estimate the time spent in moderate-to-vigorous physical activity (MVPA) in children and choice of an appropriate CoP primarily remains a subjective decision. Therefore, this study aimed to analyze the influence of CoP selection on the mean MVPA and to define the optimal thresholds of MVPA derived from different accelerometer CoPs to avoid overweight/obesity and adiposity in children aged 7 to 12 years. Three hundred six children participated. Physical activity (PA) was monitored for seven consecutive days using an ActiGraph accelerometer (model GT3X) and the intensity of PA was estimated using the five most frequently published CoPs. Body adiposity was assessed using a multi-frequency bioelectrical impedance analysis. There was found a wide range of mean levels of MVPA that ranged from 27 (Puyau CoP) to 231 min∙d^–1^ (Freedson 2005 CoP). A receiver operating characteristic curve analysis indicated that the optimal thresholds for counts per minute (cpm) and MVPA derived from the Puyau CoP was the most useful in classifying children according to their body mass index (BMI) and fat mass percentage (FM%). In the total sample, the optimal thresholds of the MVPA derived from the Puyau CoP were 22 and 23 min∙d^–1^ when the categories based on BMI and FM%, respectively, were used. The children who did not meet these optimal thresholds had a significantly increased risk of being overweight/obese (OR = 2.88, *P* < 0.01) and risk of having excess fat mass (OR = 2.41, *P* < 0.01). In conclusion, the decision of selecting among various CoPs significantly influences the optimal levels of MVPA. The Puyau CoP of 3 200 cmp seems to be the most useful for defining the optimal level of PA for pediatric obesity prevention.

## Introduction

There is a worldwide agreement that children should participate in at least 60 min of moderate-to-vigorous physical activity (MVPA) daily to achieve substantial health benefits [1–4]. Following this physical activity (PA) recommendation may improve cardiorespiratory and muscular fitness, bone health, and cardiovascular and metabolic health biomarkers; may reduce symptoms of anxiety and depression [2]; and may be appropriate to prevent childhood obesity [5–7].

Accelerometers provide several advantages compared with self-report methods in children [8, 9]; thus, they have become the most widely used method for estimating the amount of time spent in different intensities of free-living PA [10] and have been well validated in children based on a range of outcomes [11, 12]. One of the most widely used devices to objectively assess PA in research is the hip-worn accelerometer ActiGraph [9, 10].

Considerable work has been conducted to develop a cut-off point (CoP) that defines the count per minute (cpm) threshold for moderate-intensity PA (i.e., 3 METs), which resulted in the development of numerous equations to predict energy expenditure from ActiGraph-based counts [13, 14]. Unfortunately, no general consensus exists regarding the most acceptable CoP, and the choice of an appropriate CoP remains highly subjective. According to Cain et al. [13], the CoPs for calculating MVPA in children and youth range from 400 to 3 600 cpm.

The variability of accelerometer-based CoPs leads to methodological challenges in the interpretation of PA results. Strong scientific evidence shows that the selection of CoP significantly influences the mean MVPA and the proportion of children who meet the PA recommendation even though the estimate is based on identical raw accelerometer data (i.e., counts) [15–17]. As a result, a meaningful comparison between the findings of various studies is extremely difficult and could be significantly biased. This issue was defined as ‘CoP non-equivalence’ [18] and a conversion system for main CoP sets has been created as one of the possible solution for synthesizing accelerometer-derived MVPA [19].

Additionally, inconsistency in CoP selection may cause a mismatch between studies describing the relationship between PA and health outcomes such as adiposity [20]. One potential approach to clarify this issue is to determine the power with which different CoPs classify participants according to their weight status or even better to their proportion of fat mass (FM). Despite the fact that the diagnostic accuracy of widely used accelerometer-based CoPs was evaluated for body mass index (BMI) [21], there is no study comparing the accuracy of different sets of CoPs for classifying children according to their proportion of FM. Therefore, the aims of this study were 1) to compare the mean MVPA derived from the most frequently used accelerometer CoPs for children and the proportion of children who meet the current PA recommendation, and 2) to define the optimal thresholds of MVPA derived from different accelerometer CoPs to avoid overweight/obesity and adiposity in children aged 7 to 12 years. We hypothesized that the mean MVPA derived from different accelerometer CoPs differ, as well as the proportion that meet the current PA recommendation. If the optimal threshold of the MVPA established by a receiver operating characteristic (ROC) curve analysis for each accelerometer CoP will be used, the proportion of individuals who meet the optimal thresholds of MVPA will be approximately equal, as well as the likelihood of being overweight/obese and having excess FM.

## Methods

### Participants

The present study included 632 children (345 girls and 287 boys) aged 7–12 years from the eastern region of the Czech Republic. To ensure a representative sample, we randomly selected 24 elementary schools in cities with various numbers of inhabitants. The selection did not include sports schools (sports academies) or schools for pupils with special educational needs. Eight elementary schools participated in the research. The main inclusion criteria were participant age and good health condition, which was reported by their parents. Those children whose parents reported medical complications that could affect PA and body composition assessment were excluded from study. Basic information regarding the objectives and content of the research study were presented to the parents using an information booklet. The parents were also provided with a telephone number to inquire about additional information or to clarify the objective and extent of the study. We addressed approximately 1 600 parents, and 620 parents agreed to let their children participate in the research.

The study was approved by the Institutional research ethics committee of the Faculty of Physical Culture of Palacký University Olomouc (reference number 53/2012; 18 December 2012). The ethical principles of the 1964 Declaration of Helsinki and its later amendments were adhered to throughout this research. All parents provided written consent for their children to participate in this study.

### Physical activity measures

PA was monitored for seven consecutive days using a hip-worn accelerometer, ActiGraph model GT3X (ActiGraph, LLC., FL, USA), which has been considered sufficiently valid in children [22]. Information regarding the correct wearing of the accelerometer was explained prior to the initial attachment; this information was provided to the parents in written form to ensure they could check and correct the attachment on a regular basis. The accelerometers were provided to the children immediately after completing the anthropometric and FM measurements. To eliminate seasonal fluctuations in PA, the study was performed in two relatively similar seasons (spring and autumn) in 2013 and 2014.

Prior to testing, each accelerometer was calibrated according to the manufacturer’s recommendations. The time sampling interval was set to 60 s, which has been the most frequently used epoch in the literature [13], and it was used in a majority of the original calibration studies. The MVPA was calculated from the raw accelerometer data (i.e., counts) using the five predominately used accelerometer CoPs for children [13]. The following accelerometer CoPs for calculating the MVPA were used: Freedson 2005 CoP of 500 cpm [23], Freedson 1997 CoP of 706–1 263 cpm ([24]; age-dependent CoP for 3 METs), 2 000 cpm used in the European Youth Heart Study (EYHS) [25, 26], Evenson CoP of 2 296 cpm [27], and Puyau CoP of 3 200 cpm [28].

The software MeterPlus, version 4.3 (www.meterplussoftware.com) was used to screen and clean the accelerometer data files of the seven days of measurement. The non-wear time was defined as 60 min of consecutive zero counts in the vertical axis allowing 2 min of non-zero interruptions [29]. The minimum daily wearing time was set to 10 hours on weekdays and 8 hours on weekend days considering the different sleep patterns during weekends [30]. Data were considered valid if at least 5 days, including one weekend day, of recoded activity were obtained. Three hundred twenty-six children were excluded from the analysis. These children did not achieve the wearing time criteria or their data could not be assessed because of technical failures during downloading or the loss of the device. Therefore, the final sample consisted of 306 children (175 girls and 131 boys). The basic characteristics of the study sample are shown in Table 1.

**Table 1:** Descriptive characteristics of the study sample.

		Total (*N* = 306)		Girls (*N* = 175)		Boys (*N* = 131)	
		Mean	SD	Mean	SD	Mean	SD
	Age, *years*	9.8	1.3	9.7	1.3	9.9	1.2
	Height, *cm*	142.3	8.8	141.7	8.8	143.2	8.8
	Weight, *kg*	35.5	8.1	34.8	8.1	36.3	8.1
	Body mass index, *kg∙m*^*–2*^	17.3	2.5	17.2	2.5	17.5	2.5
	Fat mass^a^, *%*	16.8	7.5	17.9*	7.3	15.3	7.5
	Overweight/obesity, *%*	16		15		18	
**Physical activity**							
	Valid days^b^	6	2	6	2	6	2
	Total wear time, *min*	75	12	75	11	75	12
	Counts per minute^a^	605	134	617	137	588	128
	MVPA by Freedson et al. (2005)^a^, *min∙d*^*–1*^	231	46	234	46	225	46
	MVPA by Freedson et al. (1997)^a^, *min∙d*^*–1*^	148	42	150	43	144	42
	MVPA by EYHS^a^, *min∙d*^*–1*^	67	20	67	19	65	20
	MVPA by Evenson et al. (2008)^a^, *min∙d*^*–1*^	54	17	54	17	52	18
	MVPA by Puyau et al. (2002)^a^, *min∙d*^*–1*^	27	12	27	12	25	11

EYHS–European Youth Heart Study, MVPA–moderate-to-vigorous physical activity, SD–standard deviation.

*Significantly different from boys, independent samples *t*-test, *P*<0.01.

^a^Values were transformed to natural logarithms prior to analysis.

^b^Expressed as the median and interquartile range.

### Anthropometric and adiposity measures

The standing height was measured prior to the FM measurement using a standard procedure with an accuracy of 0.1 cm via an Anthropometer P-375 (Trystom, Olomouc, Czech Republic). The body weight was measured using the body composition analyzer InBody 720 (Biospace Co., Ltd., Seoul, Korea) with an accuracy of 0.1 kg. Sex- and age-standardized BMI CoPs by the International Obesity Task Force [31] were used to categorize the participants as non-overweight and overweight/obese.

The FM was assessed with a multi-frequency bioelectrical impedance analysis method using the manufacturer’s equation. An InBody 720 device is considered highly precise for measuring FM in children [32]. The InBody 720 device measures resistance in broadband frequencies (1, 5, 50, 250, 500 and 1 000 kHz) and reactance in mean frequencies (5, 50 and 250 kHz). The total body impedance values were calculated by summing the segmental impedance values, which were independently analyzed with a tetrapolar eight-point tactile electrode system. In accordance with the manufacturer’s guidelines, the measurement was performed in a standing position while the participant was barefoot and wearing light indoor clothing. The children were instructed in advance regarding the recommendations to observe for a period initiated 48 hours prior to the measurement to maintain the examination validity. The procedure required approximately two minutes, and it did not require specific skills. One field worker performed the body composition measurement on school premises. We used the sex-specific 85^th^ percentile of the FM percentage (FM%) to differentiate the participants with a normal FM and the participants with an excess FM (i.e., adiposity).

### Data analysis

Data were analyzed using SPSS software, version 21 (SPSS for Windows; SPSS, Chicago, IL). All relevant data are available in Supporting Information file S1 Dataset. The descriptive statistics of the outcome measures are presented as the mean and standard deviation unless otherwise stated. Sex differences were analyzed with Chi-square test and independent samples *t*-test for categorical and continuous variables, respectively. As a result of the skewed nature of the FM%, cpm and MVPA derived from different accelerometer CoPs, the natural logarithm was used in the analysis of sex differences. We calculated the mean difference in the MVPA and the 95% limits of agreement (mean difference ± 1.96 standard deviation) for all accelerometer CoP pairs. The significance of the differences between each MVPA pair were tested against zero using a one-sample *t*-test.

The ROC curve analysis was used to identify the optimal thresholds for the cpm and MVPA derived from different CoPs that best discriminate between the BMI (non-overweight and overweight/obesity) and FM% (normal FM and adiposity) categories. The optimal threshold was defined as the value of the MVPA at which the sum of the square distance between the ideal test and the sensitivity and specifıcity was minimized (i.e., *d*^2^). This method of using the lowest *d*^2^ is considered one of the two best methods for determining optimal threshold from a ROC curve analysis [33]. Sensitivity represents the probability of correctly identifying overweight/obesity and adiposity, respectively. Specificity represents the probability of correctly identifying non-overweight and children with a normal FM, respectively. The ROC curve accuracy was measured by the area under the curve (AUC), in which the AUC values range from 0.5 (worthless test) to 1 (ideal test).

A contrasting group analysis [34] was used to evaluate the specific discriminative capability of optimal thresholds derived from a ROC curve analysis. The optimal threshold that aggregated the highest probability of correct decisions, the lowest misclassification errors, the highest validity coefficient, the highest expected utility, the lowest expected disutility, and the highest expected maximal utility was deemed to be the best for discriminating between BMI and FM% categories, respectively.

The likelihood of overweight/obesity and an excess FM (dependent variables) in children who do not meet the optimal thresholds (independent variables) was estimated using a binary logistic regression analysis. The results of the regression analysis are presented as the odds ratio (OR) and corresponding 95% confidence interval. All analyses were 2-tailed and were performed with the alpha value defined as 0.05.

## Results

The descriptive characteristics of the participants are presented in Table 1. The mean age was 9.8 ± 1.3 years, the height was 142.3 ± 8.8 cm, the weight was 35.5 ± 8.1 kg and the BMI was 17.3 ± 2.5 kg∙m^–2^; these characteristics did not differ between sexes. Although we did not find a significant association between sex and weight status (*χ*^2^ = 0.41; *P* = 0.52), the girls had a significantly increased FM% by 2.6% (*P* < 0.01) compared with the boys. The mean cpm and MVPA derived from the different CoPs were increased in the girls; however, significant differences between the sexes were not identified. A wide range of mean MVPA derived from different CoPs was identified, which ranged from 27 (Puyau CoP) to 231 min∙d^–1^ (Freedson 2005 CoP) in the total sample. The mean differences between the MVPA derived from the different CoPs and the 95% limits of agreements are shown in Table 2. We identified significant differences between the MVPA for all CoP pairs (all *P* < 0.01). The least mean difference of 13±4 min∙d^–1^ was identified between the MVPA derived from the EYHS and Evenson CoPs.

**Table 2:** Differences in the minutes of moderate-to-vigorous physical activity per day derived from different cut-off points in children.

	Total (*N* = 306)					Girls (*N* = 175)					Boys (*N* = 131)				
	Mean	SD	95% LoA			Mean	SD	95% LoA			Mean	SD	95% LoA		
Freedson 2005 *vs*. Freedson 1997	83	20	44	to	122	84	20	45	to	123	81	20	42	to	120
Freedson 2005 *vs*. EYHS	164	36	93	to	235	167	35	98	to	236	160	36	89	to	231
Freedson 2005 *vs*. Evenson	177	38	103	to	251	180	38	106	to	254	173	39	97	to	249
Freedson 2005 *vs*. Puyau	204	43	120	to	288	207	43	123	to	291	200	44	114	to	286
Freedson 1997 *vs*. EYHS	81	32	18	to	144	83	33	18	to	148	79	31	18	to	140
Freedson 1997 *vs*. Evenson	94	35	25	to	163	96	35	27	to	165	92	34	25	to	159
Freedson 1997 *vs*. Puyau	121	40	43	to	199	123	40	45	to	201	119	39	43	to	195
EYHS *vs*. Evenson	13	4	5	to	21	13	4	5	to	21	13	4	5	to	21
EYHS *vs*. Puyau	40	12	16	to	64	40	12	16	to	64	40	13	15	to	65
Evenson *vs*. Puyau	27	9	9	to	45	27	8	11	to	43	28	9	10	to	46

EYHS–European Youth Heart Study, LoA–limits of agreement, SD–standard deviation.

All differences are significantly different from zero, one sample *t*-test, *P*<0.01.

**Fig 1:**
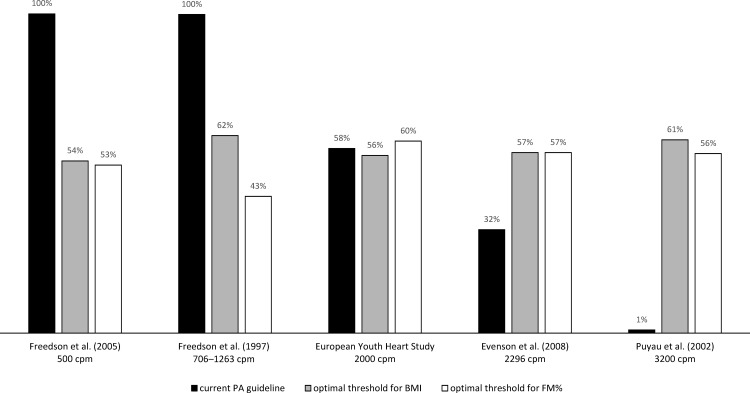
Proportion of children who meet the current PA recommendation and the recommended level of moderate-to-vigorous physical activity derived from the ROC curve analysis for each cut-off point.

In the case of the mean MVPA derived from different CoPs, there were noticeable differences in the proportion of children who met the current PA recommendation (Fig 1). All children met the current PA recommendation when the Freedson 1997 and Freedson 2005 CoPs were applied; however, only 3 of 306 children performed ≥60 min∙d^–1^ of MVPA derived from the Puyau CoP. Thus, we constructed ROC curves to determine the optimal MVPA threshold to identify overweight/obese children and children with an excess FM for all CoPs. We hypothesized that the proportion of children who meet the optimal thresholds will be approximately equal across all CoPs.

The ROC curve analysis using the BMI categories indicated that the cpm and MVPA derived from different CoPs discriminated among non-overweight and overweight/obesity, with the exception of the MVPA derived from Freedson 2005 (*P* = 0.05) and Freedson 1997 (*P* = 0.27) CoPs in girls (Table 3). According to a contrasting group analysis, the optimal threshold for cpm seems to be the best for discriminating between BMI categories in the total sample and both sexes. Additionally, the best discriminative ability among the five selected CoPs has been shown for the optimal thresholds derived from the Puyau CoP. The significant thresholds of MVPA derived from the Puyau CoP associated with non-overweight were 22 min∙d^–1^ (*P* < 0.01) in the total sample, 24 min∙d^–1^ (*P* < 0.01) in the girls and 17 min∙d^–1^ (*P* = 0.02) in the boys.

**Table 3:** Evaluation of discriminative ability of optimal thresholds to identify overweight/obesity for counts per minute and moderate-to-vigorous physical activity derived from different cut-off points in children.

		Optimal threshold	Se (%)	Sp (%)	AUC	*P*-value	Probability of correct decision	Misclassification errors (Type I/Type II)	Validity coefficient	EU	ED	EMU
**Total (*N* = 306)**												
	Counts per minute	574	71	62	0.69	<0.01	0.64	0.32/0.05	0.25	0.75	–0.41	105
	MVPA by Freedson et al. (2005), *min∙d*^*–1*^	221	67	57	0.65	<0.01	0.59	0.36/0.06	0.17	0.69	–0.47	69
	MVPA by Freedson et al. (1997), *min∙d*^*–1*^	129	53	65	0.61	0.01	0.63	0.30/0.08	0.13	0.71	–0.45	81
	MVPA by EYHS, *min∙d*^*–1*^	61	65	60	0.67	<0.01	0.60	0.34/0.06	0.18	0.71	–0.45	79
	MVPA by Evenson et al. (2008), *min∙d*^*–1*^	49	65	60	0.67	<0.01	0.61	0.33/0.06	0.17	0.71	–0.45	79
	MVPA by Puyau et al. (2002), *min∙d*^*–1*^	22	61	66	0.67	<0.01	0.64	0.30/0.06	0.19	0.74	–0.42	97
**Girls (*N* = 175)**												
	Counts per minute	578	65	63	0.64	0.02	0.63	0.18/0.05	0.35	0.73	–0.28	79
	MVPA by Freedson et al. (2005), *min∙d*^*–1*^	222	58	60	0.62	0.05	0.59	0.20/0.06	0.27	0.68	–0.32	63
	MVPA by Freedson et al. (1997), *min∙d*^*–1*^	129	50	69	0.57	0.27	0.65	0.16/0.07	0.27	0.73	–0.31	67
	MVPA by EYHS, *min∙d*^*–1*^	61	62	64	0.65	0.02	0.62	0.18/0.06	0.32	0.71	–0.30	73
	MVPA by Evenson et al. (2008), *min∙d*^*–1*^	49	62	64	0.66	0.01	0.63	0.18/0.06	0.30	0.71	–0.30	72
	MVPA by Puyau et al. (2002), *min∙d*^*–1*^	24	69	59	0.68	<0.01	0.58	0.22/0.04	0.36	0.69	–0.30	68
**Boys (*N* = 131)**												
	Counts per minute	542	78	64	0.74	<0.01	0.66	0.13/0.05	0.51	0.79	–0.22	74
	MVPA by Freedson et al. (2005), *min∙d*^*–1*^	221	78	54	0.67	0.01	0.58	0.16/0.04	0.48	0.72	–0.24	63
	MVPA by Freedson et al. (1997), *min∙d*^*–1*^	116	52	73	0.66	0.02	0.69	0.10/0.08	0.39	0.78	–0.27	67
	MVPA by EYHS, *min∙d*^*–1*^	59	70	59	0.68	0.01	0.60	0.14/0.06	0.42	0.72	–0.27	59
	MVPA by Evenson et al. (2008), *min∙d*^*–1*^	44	61	68	0.69	0.01	0.66	0.11/0.07	0.43	0.77	–0.25	68
	MVPA by Puyau et al. (2002), *min∙d*^*–1*^	17	57	79	0.66	0.02	0.74	0.08/0.08	0.44	0.83	–0.24	77

AUC–area under the curve, ED–expected disutility, EMU–expected maximal utility, EU–expected utility, EYHS–European Youth Heart Study, MVPA–moderate-to-vigorous physical activity, Se–sensitivity, Sp–specificity.

While the ROC curve analysis using the FM% categories was performed in the total sample, the AUCs were significantly greater than 0.5 in the cpm and all MVPA with the exception of the Freedson 1997 CoP (*P* = 0.05) (Table 4). In the girls, only the MVPA derived from the Puyau CoP (*P* = 0.01) discriminated among the FM% categories. In the boys, all MVPA discriminated among the FM% categories with the exception of the MVPA derived from the EYHS (*P* = 0.07) and Puyau (*P* = 0.07) CoPs. In the total sample, the optimal threshold for cpm showed the best discriminate ability with the highest probability of correct decisions (0.62), the lowest misclassification errors (0.33/0.05), the highest validity coefficient (0.2), the highest expected utility (0.72), the lowest expected disutility (–0.43), and the highest expected maximal utility (89) in comparison with MVPA derived from selected CoPs. A contrasting group analysis showed that the optimal threshold for MVPA derived from the Puyau CoP is the most useful in classifying children according to their FM% in comparison with the other four CoPs.

**Table 4:** Evaluation of discriminative ability of optimal thresholds to identify adiposity for counts per minute and moderate-to-vigorous physical activity derived from different cut-off points in children.

		Optimal threshold	Se (%)	Sp (%)	AUC	*P*-value	Probability of correct decision	Misclassification errors (Type I/Type II)	Validity coefficient	EU	ED	EMU
**Total (*N* = 306)**												
	Counts per minute	573	67	61	0.65	<0.01	0.62	0.33/0.05	0.20	0.72	–0.43	89
	MVPA by Freedson et al. (2005), *min∙d*^*–1*^	222	64	57	0.63	0.01	0.57	0.38/0.05	0.14	0.66	–0.48	55
	MVPA by Freedson et al. (1997), *min∙d*^*–1*^	149	71	45	0.59	0.05	0.49	0.47/0.04	0.12	0.59	–0.55	13
	MVPA by EYHS, *min∙d*^*–1*^	59	59	62	0.61	0.02	0.61	0.32/0.07	0.10	0.69	–0.46	69
	MVPA by Evenson et al. (2008), *min∙d*^*–1*^	49	62	59	0.62	0.01	0.59	0.35/0.06	0.14	0.68	–0.46	67
	MVPA by Puyau et al. (2002), *min∙d*^*–1*^	23	64	59	0.65	<0.01	0.60	0.35/0.06	0.15	0.69	–0.46	71
**Girls (*N* = 175)**												
	Counts per minute	573	54	65	0.59	0.14	0.63	0.17/0.07	0.28	0.71	–0.31	71
	MVPA by Freedson et al. (2005), *min∙d*^*–1*^	212	42	73	0.59	0.15	0.69	0.13/0.09	0.24	0.75	–0.30	78
	MVPA by Freedson et al. (1997), *min∙d*^*–1*^	107	35	87	0.54	0.55	0.79	0.07/0.10	0.30	0.84	–0.26	102
	MVPA by EYHS, *min∙d*^*–1*^	63	58	58	0.60	0.12	0.58	0.21/0.06	0.26	0.66	–0.33	58
	MVPA by Evenson et al. (2008), *min∙d*^*–1*^	49	62	64	0.62	0.06	0.63	0.18/0.06	0.30	0.71	–0.30	72
	MVPA by Puyau et al. (2002), *min∙d*^*–1*^	20	54	75	0.66	0.01	0.71	0.13/0.07	0.34	0.79	–0.26	92
**Boys (*N* = 131)**												
	Counts per minute	542	79	63	0.73	<0.01	0.64	0.14/0.04	0.47	0.75	–0.21	70
	MVPA by Freedson et al. (2005), *min∙d*^*–1*^	221	84	54	0.67	0.02	0.58	0.17/0.02	0.50	0.70	–0.22	64
	MVPA by Freedson et al. (1997), *min∙d*^*–1*^	149	90	46	0.65	0.04	0.52	0.20/0.02	0.50	0.65	–0.23	55
	MVPA by EYHS, *min∙d*^*–1*^	53	53	71	0.63	0.07	0.68	0.11/0.07	0.36	0.76	–0.25	67
	MVPA by Evenson et al. (2008), *min∙d*^*–1*^	44	58	68	0.64	0.05	0.65	0.12/0.06	0.38	0.73	–0.25	64
	MVPA by Puyau et al. (2002), *min∙d*^*–1*^	17	53	77	0.63	0.07	0.73	0.08/0.08	0.37	0.79	–0.24	73

AUC–area under the curve, ED–expected disutility, EMU–expected maximal utility, EU–expected utility, EYHS–European Youth Heart Study, MVPA–moderate-to-vigorous physical activity, Se–sensitivity, Sp–specificity.

The proportions of children who met the optimal threshold of the MVPA established by the ROC curve analysis for each CoP are shown in Fig 1. The proportion of children who met the optimal MVPA thresholds ranged from 54 to 62% and 43 to 60% when the optimal thresholds were established for the BMI and FM% categories, respectively. In the case of the EYHS CoP, we identified negligible differences in the proportion of children who met the current PA recommendation and the children who met the optimal thresholds of the MVPA derived from the ROC curve analysis.

**Fig 2:**
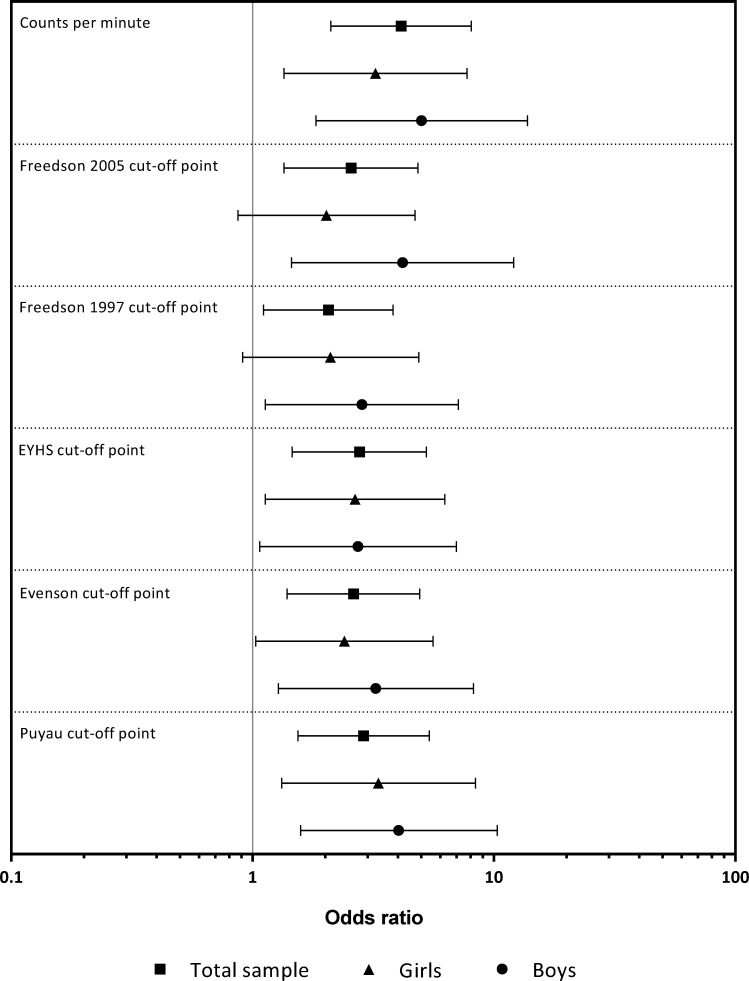
Odds ratios of being overweight/obese in children who did not meet the counts per minute and moderate-to-vigorous physical activity threshold derived from the ROC curve analysis for each cut-off point.

Fig 2 compares the ORs of being overweight/obese if the children did not meet the optimal threshold for the cpm and MVPA in the total sample, as well as for both sexes. The children who did not meet the cpm and MVPA optimal thresholds for all CoPs had a significantly increased risk of being overweight/obese with the exception of the girls who did not meet the optimal threshold from the Freedson 2005 CoP (OR = 2.02, *P* = 0.10) and the Freedson 1997 CoP (OR = 2.10, *P* = 0.08).

**Fig 3:**
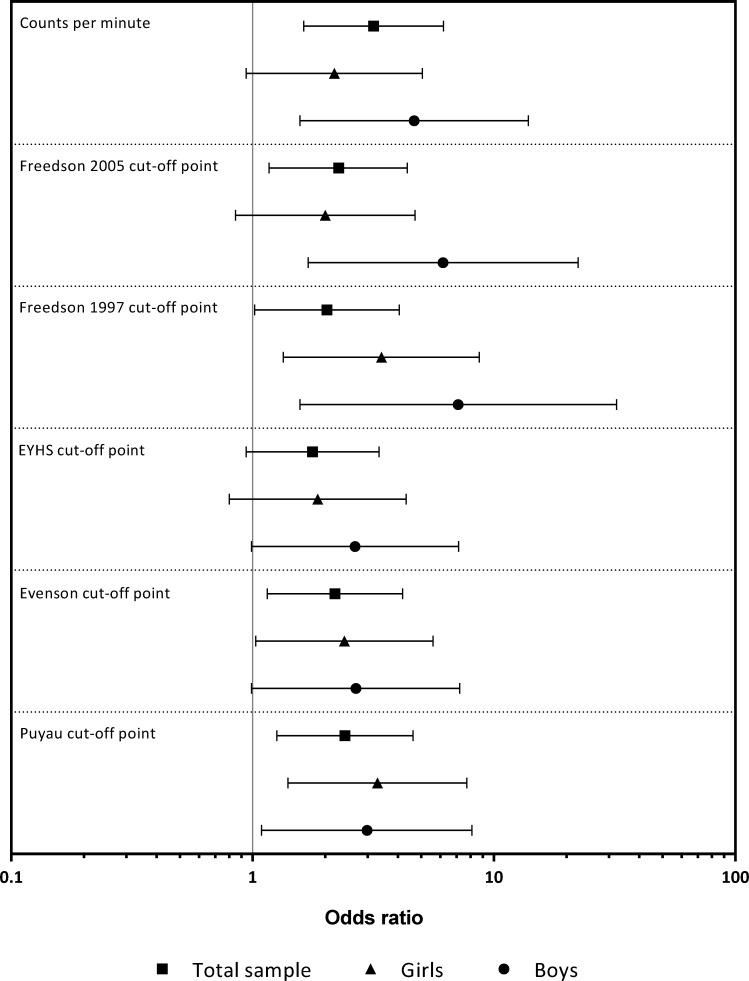
Odds ratios of having excess fat mass in children who did not meet the counts per minute and moderate-to-vigorous physical activity threshold derived from the ROC curve analysis for each cut-off point.

Fig 3 shows the ORs of having excess FM if the children did not meet the cpm and MVPA optimal thresholds. In the total sample, the ORs for having excess FM if the children did not meet the optimal threshold were significant for the cpm (OR = 3.17, *P* < 0.01), Freedson 2005 CoP (OR = 2.27, *P* = 0.02), Freedson 1997 CoP (OR = 2.03, *P* = 0.04), Evenson CoP (OR = 2.19, *P* = 0.02) and Puyau CoP (OR = 2.41, *P* < 0.01). The girls who did not meet 107 min∙d^–1^ (OR = 3.42, *P* = 0.01), 49 min∙d^–1^ (OR = 2.40, *P* = 0.04) and 20 min∙d^–1^ (OR = 3.29, *P* = 0.01) derived from the Freedson 1997, Evenson and Puyau CoPs, respectively, had an increased risk of having excess FM. Furthermore, the boys who did not meet 221 min∙d^–1^ (OR = 6.15, *P* < 0.01), 149 min∙d^–1^ (OR = 7.11, *P* = 0.01) and 17 min∙d^–1^ (OR = 2.98, *P =* 0.03) derived from the Freedson 2005 CoP, Freedson 1997 CoP and Puyau CoP, respectively, had an increased risk of having excess FM.

## Discussion

The present study analyzed the discrepancies in the ActiGraph accelerometer outputs defined using five sets of CoPs with the risk of being overweight/obese and adiposity in children. The study has three main findings. First, the study confirmed that the mean levels of MVPA significantly differ depending on the algorithms applied to the raw data conversion. Second, an objectively assessed level of MVPA may be used as a decision making method to identify overweight/obese children and children with excess FM, which was confirmed by the ROC curve analysis. The optimal thresholds of MVPA derived from the Puyau CoP showed the best discriminative ability and was the most useful in classifying children according to their BMI and FM% in comparison with the other four CoPs. Finally, the proportions of children who meet the current PA recommendation significantly vary considering CoP use; therefore, it is not possible to use one general threshold of MVPA for all CoPs. Thus, the present study provides a set of optimal thresholds of raw accelerometer data (i.e., cpm) and MVPA derived from different CoPs. This approach leads to unification in the proportion of children who meet the recommended levels of MVPA estimated by the ROC curve analysis, as well as unification of the likelihood of being overweight/obese and having excess FM, if the optimal threshold of MVPA is not met.

Our findings of a wide range of mean MVPA derived from different CoPs is in agreement with previous studies in preschool children [18], school children and adolescents [15, 16, 35]. In terms of the proportion of children who meet the current PA recommendation, the Puyau, Freedson 2005 and Freedson 1997 CoPs appear to not be suitable for the assessment of PA in children because 1 or 100% of the participants met the current PA recommendation. These findings correspond with several studies that emphasize the proportion of children and adolescents who meet the current PA recommendation ranges between 3 and 100%, depending on the CoPs used for MVPA estimation [16, 36].

The optimal thresholds for MVPA derived from the Puyau CoP demonstrated the highest discriminative ability to classify children according to their BMI and FM% than the other four CoPs. Our results support the findings by Guinhouya et al. [21] who presented that MVPA derived from the Puyau CoP had a higher discriminative ability to classify overweight/obesity than MVPA derived from lower CoPs (i.e., <3 200 cpm). However, the optimal thresholds of MVPA derived from the Puyau CoP were approximately threefold lower than the current PA recommendation of ≥60 min∙d^–1^. On the other hand, the optimal thresholds for the EYHS CoP were the closest to the current PA recommendation. Similar findings have been reported by Laguna et al. [6] who demonstrated that the significant threshold for identifying non-overweight children was 67 min∙d^–1^ of MVPA derived from the EYHS CoP. Although the optimal thresholds of MVPA derived from the EYHS CoP were the closest to the current PA recommendation, its discriminative ability was lower compared with the Puyau CoP.

BMI has a low sensitivity in the identification of adiposity in children [37]; thus, we present a set of optimal thresholds that best discriminate between children with a normal FM and children with excess FM measured using a multi-frequency bioelectrical impedance analysis. Compared with the BMI categories, the results exhibited greater differences between sexes when the FM% categories were used. Poor diagnostic performances of MVPA represented by the AUCs were identified particularly in girls. In girls, only MVPA derived from the Puyau CoP exhibited the significant discriminative ability to classify adiposity. This finding may indicate the influence of sex on the association between MVPA and FM in children. A negative association between MVPA and FM has been previously demonstrated in both sexes [25, 38]; however, Ness et al. [39] emphasized that the association is stronger in boys compared with girls.

We agree that for interpretations of PA recommendations for public health and policy makers, it is more appropriate to use a simple message, such as a number (i.e., 60 minutes), compared with a recommendation expressed by the cpm. However, because of the inconsistencies across studies, researchers must be careful particularly in implementing the findings into recommendations for public health practice. In our study, we calculated the optimal threshold for each CoP, which may serve as a conversion table between different sets of CoPs. The cpm has a comparable diagnostic performance with the MVPA derived from different CoPs; thus, we agree with the statement of Basset and colleagues [9] that one potential approach that enables comparisons across studies is to present raw data related to the time of wearing, such as the total activity counts.

To the best of our knowledge, this study is the first investigation to demonstrate the effect of CoPs on the recommended level of PA in the prevention of both overweight/obesity and adiposity in children. The strengths of the present study include the objective measure of PA by ActiGraph accelerometers, which are recognized as the most widely used device to assess PA in research. Thus, our study has the potential to be of interest to many readers. There are also several limitations that should be considered when applying these results in future research. First, we did not use a reference method for FM measurement, which may have resulted in bias; however, several studies have demonstrated a high precision of multi-frequency bioelectrical impedance analysis to estimate FM in children [32, 40]. Second, we were not able to calculate the individual energy expenditure of the participants. This underestimates the energy expenditure during activity of the children who had greater body weights. Third, the moderate parental response rate may have resulted in a selection bias. Mellor, Rapoport and Maliniak [41] reported that parents of children who are overweight or at risk of being overweight are significantly less likely to give active consent. This could explain the slightly lower prevalence of overweight and obesity than was estimated for Czech youth [42]. Finally, the methodological issues include the different sets of CoPs, as well as other parameters, such as the time sampling (epoch length), the definition of non-wear time within a day, the definition of valid day and the decisions that define the minimum number of valid days. These issues must be addressed in future research.

## Conclusions

In conclusion, the present study demonstrates that the decision of CoP selection has a significant influence on the estimates of MVPA, and it strongly affects the relative possibility of participants to meet the PA recommendation. A contrasting group analysis showed that the optimal thresholds of MVPA derived from the Puyau CoP showed the best discriminative ability and was the most useful in classifying children according to their BMI and FM% in comparison with the other four CoPs. With experience from previous studies, we encourage the major scientific organizations that deal with PA to establish general guidelines for publishing accelerometer derived data. The current, inconsistent methodologies across studies may decrease the value of scientific work and may negatively influence the prestige of the research community, especially regarding the implementation of findings into recommendations for public health practice and the presentation of results to policy makers and the general public.

## Supporting Information

S1 Dataset Original data. This file containing original data.
(SAV)
